# Gamma analysis dependence on specified low‐dose thresholds for VMAT QA

**DOI:** 10.1120/jacmp.v16i6.5696

**Published:** 2015-11-08

**Authors:** Ji‐Hye Song, Min‐Joo Kim, So‐Hyun Park, Seu‐Ran Lee, Min‐Young Lee, Dong Soo Lee, Tae Suk Suh

**Affiliations:** ^1^ Department of Biomedical Engineering and Research Institute of Biomedical Engineering College of Medicine, The Catholic University of Korea Seoul 137‐701 Korea; ^2^ Department of Radiation Oncology Uijeongbu St. Mary's Hospital Uijeongbu 480‐717 Korea

**Keywords:** Gamma analysis, Low dose threshold, VMAT, EPID, QA

## Abstract

The American Association of Physicists in Medicine Task Group 119 instructed institutions to use a low‐dose threshold of 10% or a region of interest determined by the jaw setting when they collected gamma analysis quality assurance (QA) data for the planar dose distribution. However, there are no clinical data to quantitatively demonstrate the impact of the low‐dose threshold on the gamma index. Therefore, we performed a gamma analysis with various low‐dose thresholds in the range of 0% to 15% according to both global and local normalization and different acceptance criteria (3%/3 mm, 2%/2 mm, and 1%/1 mm). A total of 30 treatment plans — 10 head and neck, 10 brain, and 10 prostate cancer cases — were randomly selected from the Varian Eclipse treatment planning system (TPS). For the gamma analysis, a calculated portal image was acquired through a portal dose calculation algorithm in the Eclipse TPS, and a measured portal image was obtained using an electronic portal‐imaging device. Then, the gamma analysis was performed using the Portal Dosimetry software (Varian Medical Systems, Palo Alto, CA). The gamma passing rate (%GP) for the global normalization decreased as the low‐dose threshold increased, and all low‐dose thresholds led to %GP values above 95% for both the 3%/3 mm and 2%/2 mm criteria. However, for the local normalization, %GP for a low‐dose threshold of 10% was 7.47%, 10.23%, and 6.71% greater than the low‐dose threshold of 0% for head and neck, brain, and prostate for the 3%/3 mm criteria, respectively. The results indicate that applying the low‐dose threshold to global normalization does not have a critical impact on patient‐specific QA results. However, in the local normalization, the low‐dose threshold level should be carefully selected because the excluded low‐dose points could cause the average %GP to increase rapidly.

PACS number: 87.55.Qr

## I. INTRODUCTION

Volumetric‐modulated arc therapy (VMAT) is utilized in clinics because of its enhanced tumor control, low toxicity to the organs at risk, and efficient treatment delivery.^1^, ^2^ The enhanced flexibility of VMAT is achieved by applying a continuous modulation of factors such as the gantry speed, dose rate, and dynamic movement of multileaf collimators (MLC) and may yield a highly optimal dose distribution with a small beam delivery time.^3^, ^4^, ^5^ However, the high modulation of the beam in VMAT can cause a dose discrepancy in the beam delivery because of the disagreement between the planned and actual movements of the machine. Thus, VMAT planning requires dedicated pretreatment quality assurance (QA).^(6)^

In order to evaluate patient‐specific VMAT QA, which is a fundamental process to confirm accurate dose delivery to the patient, VMAT studies have commonly employed gamma analysis to detect possible beam delivery errors.^7^, ^8^, ^9^ Gamma analysis was first introduced by Low et al.^(10)^ to quantitatively compare calculated and measured dose distributions, and this technique uses the physical distance and dose difference, which are normalized by the acceptance criteria: the distance to agreement (DTA) and the dose differences (DD).^(11)^ Gamma analysis has been discussed extensively in many papers in terms of various criteria, the searching range, and a comparison between two‐dimensional (2D) and 3D gamma analysis. These studies compared various gamma analyses and suggested adequate levels of gamma parameters.^2^, ^3^, ^12^, ^13^

The American Association of Physicists in Medicine (AAPM) Task Group 119 (TG‐119) evaluated the overall accuracy of planning and delivery of intensity‐modulated radiation therapy (IMRT) techniques and produced quantitative confidence limits as a result. The TG‐119 instructed facilities to use a low‐dose threshold of 10% or a region of interest (ROI) determined by the jaw setting when they collected gamma analysis QA data on planar dose distributions.^(14)^ The low‐dose threshold is a parameter to exclude dose points below a selected threshold for the calculation of the gamma index. According to a survey by Nelms and Simon,^(15)^ more than 70% of clinics use a low‐dose threshold of 0% to 10% for gamma analysis. However, even TG‐119 has no clinical data to quantitatively demonstrate the impact of the low‐dose threshold on the gamma index.

Modulated radiation fields usually undergo the conventional QA process: (a) application of a patient treatment plan to the phantom and exposure of the planned fluence to the phantom; (b) comparison of the calculated and measured dose distributions by analyzing an evaluation index, such as a gamma index, a homogeneity index, or a conformity index, et cetera; and (c) decision about the treatment procedure based on the QA results, either by performing treatment without further modification or by performing an additional validation process after modification of the treatment plan.^14^, ^16^ However, recently, interest in electronic portal image device (EPID) dosimetry has increased because EPID dosimetry can reduce the labor‐intensity QA procedure with the phantom setting and ensure acquisition of a high‐resolution dose distribution in a digital format.^(17)^ Therefore, EPID dosimetry has gained increased attention over time.

Thus, the objective of our study was to quantitatively investigate the effect of the low‐dose threshold on gamma analysis according to three acceptance criteria, four different low‐dose thresholds, and two normalization methods based on EPID dosimetry of VMAT plans.

## II. MATERIALS AND METHODS

### A. Treatment plan selection

A total of 30 treatment plans — 10 head and neck, 10 brain, and 10 prostate cancer cases — were randomly selected retrospectively from the Varian Eclipse treatment planning system (TPS) (version 10.0, Varian Medical Systems, Palo Alto, CA). All of the head and neck and prostate cancer cases were planned using two full arcs. Among the brain cases, two full arcs were applied in seven cases, and one and a half arcs were applied in three cases. An optimized treatment plan containing calculated fluence and dynamic MLC leaf motions was generated using the parameters given in Table 1.

**Table 1:** Planning parameters for volumetric modulated arc therapy (VMAT)

*Anatomical Region*	*Energy (MV)*	*Prescription Dose (Gy)/Fraction*	*Number of Arcs/Range of Angles (°)*	*Algorithm*
Head and neck	6	70/35	2/360	AAA
Brain	6	59.4/33	2/360 for seven cases	AAA
			1/360+1/180 for three cases	
Prostate	10	73.8/41	2/360	AAA
		70/28		

AAA = Analytical Anisotropic Algorithm

### B. Portal dosimetry

The EPID, a Varian PortalVision aS1000, was mounted on the Exact arm of a Clinac iX linear accelerator (Varian Medical Systems) and was composed of a matrix of 1024×768 pixels with a light‐sensitive photodiode and a thin‐film transistor. It had a pixel pitch of $0.039\times 0.034\;{\text{cm}}^{2}$ with an active detector area of $40\times 30\;{\text{cm}}^{2}$.^18^, ^19^, ^20^ The EPID response was calibrated by both a dark field to correct the background signal and a flood field to adjust the variation in pixel sensitivity. Then, the EPID was scaled such that one calibrated unit corresponded to 100 monitor units of a $10\times 10\;{\text{cm}}^{2}$ field size at a source‐to‐detector distance (SDD) of 100 cm. For every VMAT plan, a portal dose verification plan was created with a SDD of 100 cm in the Eclipse TPS using a portal dose calculation (PDC) algorithm that employed a dose reconstruction approach with no patient or phantom.^18^, ^21^ The PDC algorithm, which was added a backscattered dose prediction model, was used to compensate the impact of the backscatter of the EPID arm.^(22)^ The Clinac iX linear accelerator delivered the verification dose to the EPID to acquire portal dose images with a SDD of 100 cm. Then, the measured portal images were compared with the corresponding calculated portal images using the Portal Dosimetry software (version 10.0, Varian Medical Systems) in the absolute mode. The portal dosimetry process is described in further detail elsewhere.^17^, ^19^, ^23^, ^24^

### C. Gamma analysis

Gamma analysis was performed between the calculated and measured portal images using the Portal Dosimetry software. The gamma index was calculated according to Low et al.^(10)^ Acceptance criteria of 3%/3 mm, 2%/2 mm, and 1%/1 mm were applied in the gamma analysis, and the gamma passing rate (%GP) was defined as the percentage of points satisfying the condition that the gamma index was less than one. The reference level of the %GP was 95% in this study for the following reasons: (a) the survey analysis indicated that a %GP of 90%–95% is a generally used acceptable level when institutions apply the 3%/3 mm criterion;^(15)^ and (b) a %GP that depends on three different criteria, four low‐dose thresholds, and two normalization methods can be directly evaluated according to the fixed level of the %GP. Table 2 shows all the parameters applied in the gamma analysis in this study.

**Table 2:** Parameters for gamma analysis in the Portal Dosimetry software

*Anatomical Region*	*Normalization*	*Acceptance Criteria*	*Low‐dose Threshold*
Head and neck	Global	3%/3 mm	0%
Brain	Local	2%/2 mm	5%
Prostate		1%/1 mm	10%
			15%

#### C.1. Normalization method

The normalization for the dose difference was performed by two different methods. The global normalization applied the maximal value of the calculated dose distribution. To avoid outliers, the highest value of the histogram — 0.1% — was cut off; then, the maximal value of the calculated dose was taken.^(25)^ In contrast, the local normalization employed a local value, which was the calculated dose at the currently evaluated pixel.

#### C.2. Low‐dose threshold

Low‐dose thresholds of 0%, 5%, 10%, and 15% were selected.^12^, ^15^ The dose threshold is a fraction of the maximal calculated dose, which excludes the highest value of the histogram — 0.1% — to prevent outliers.^(25)^ For the ROI in the gamma calculation, the Portal Dosimetry software provides the following options to users: none, field with/without margin, and MLC with/without margin. In our study, the ROI was determined as a field without margin. Both the low‐dose threshold and ROI setting were simultaneously applied to entire cases to exclude regions outside of the field, which are redundant dose points or regions, especially for a low‐dose threshold of 0%, even though TG‐119 suggests using either the low‐dose threshold or the ROI setting. We also performed additional gamma analysis without the ROI restriction to confirm whether the ROI setting affected our results. This extra analysis was implemented only for a low‐dose threshold of 0% because the selection of the ROI setting — none — only affected the results for the ROI as a field at this low‐dose threshold.

### D. Evaluation method

To quantitatively identify the impact of the low‐dose threshold value on the gamma analysis, we calculated the percentage change in the %GP according to the following equation:

(1) $$Percentage\;change=\left|\frac{\%GP_{x\%}-\%GP_{0\%}}{\%GP_{0\%}}\right|\times 100$$

where the $\%GP_{x\%}$ is an average of %GP with the low‐dose threshold of x%, and the $\%GP_{0\%}$ is an average of %GP with the low‐dose threshold of 0%. To verify the significant difference between $\%{\text{GP}}_{x\%}$ and $\%{\text{GP}}_{0\%}$, the nonparametric Kruskal‐Wallis test was used with a significance level of 0.05. As a posteriori test, Mann‐Whitney analysis was implemented with an adjusted significance level of 0.0083. All statistical analyses were performed using the SPSS software (version 17.0.0, SPSS Inc., Chicago, IL).

## III. RESULTS

### A. Global gamma analysis

**Figure 1:**
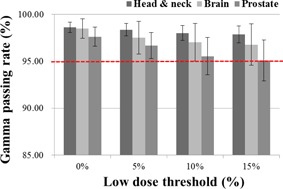
Average gamma passing rate (%GP) and standard deviation for the 2%/2 mm global gamma analysis: the dotted red line indicates a %GP of 95%. All low‐dose thresholds show an average %GP above 95%.

The average and standard deviation of the %GP for all acceptance criteria for the global normalization were calculated for each of the 30 patients and are shown in Table 3. The %GP decreased as the low‐dose threshold increased from 0% to 15%. This decrease rate was as great as 0.22%, 0.23%, and 0.16% in the head and neck, brain, and prostate cancer cases, respectively, for the 3%/3 mm criterion. When more stringent criteria were applied, this tendency was more apparent, with decreases as great as 0.74%, 1.76%, and 2.59% in the head and neck, brain, and prostate cancer cases, respectively, for the 2%/2 mm criterion. All head and neck, brain, and prostate cancer cases with the applied dose thresholds exhibited a %GP above 95% for both the 3%/3 mm and 2%/2 mm criteria (Fig. 1).

**Table 3:** Average gamma passing rate with standard deviation in global gamma analysis

*Acceptance Criteria*		*Low‐dose Threshold*			
	*Site*	*0%*	*5%*	*10%*	*15%*
3%/3 mm	H&N	99.80±0.09	99.70±0.15	99.59±0.19	99.58±0.19
	Brain	99.86±0.17	99.72±0.29	99.66±0.36	99.63±0.38
	Prostate	99.74±0.22	99.72±0.25	99.61±0.32	99.58±0.35
2%/2 mm	H&N	98.61±0.56	98.38±0.66	98.02±0.80	97.88±0.89
	Brain	98.51±1.04	97.53±1.73	97.04±2.02	96.78±2.21
	Prostate	97.62±1.01	96.67±1.39	95.55±1.98	95.09±2.18
1%/1 mm	H&N	87.24±3.84	84.01±4.40	82.97±5.22	82.32±5.62
	Brain	81.53±7.49	78.54±8.65	76.93±9.61	75.43±10.46
	Prostate	79.22±4.40	72.41±4.91	67.19±6.44	64.91±7.04

H&N=head and neck.

### B. Local gamma analysis

**Figure 2:**
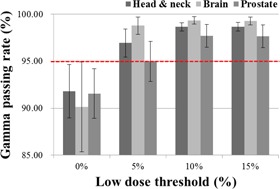
Average gamma passing rate (%GP) and standard deviation for the 3%/3 mm local gamma analysis: the dotted red line indicates a %GP of 95%. All low‐dose thresholds show an average %GP above 95%, except a low‐dose threshold of 0% in all cases and a low‐dose threshold of 5% in the prostate case.

The average and standard deviation of the %GP for all acceptance criteria for the local normalization were calculated for each of the 30 patients and are shown in Table 4. The %GP increased as the low‐dose threshold increased from 0% to 15%. This increase rate was as great as 7.50%, 10.16%, and 6.66% in the head and neck, brain, and prostate cancer cases, respectively, for the 3%/3 mm criterion. When the 2%/2 mm criterion was applied, the %GP increased as much as 15.84%, 22.28%, and 13.01% in the head and neck, brain, and prostate cancer cases, respectively. For the 3%/3 mm criterion, the average %GP was above 95% with the exception of the average %GP for low‐dose thresholds of 0% for all cases and 5% for the prostate cancer cases (Fig. 2). The average %GP for both the 2%/2 mm and 1%/1 mm criteria was below 95% for all low‐dose thresholds.

**Table 4:** Average gamma passing rate with standard deviation in local gamma analysis

*Acceptance Criteria*	*Low‐dose Threshold*				
	*Site*	*0%*	*5%*	*10%*	*15%*
3%/3 mm	H&N	91.79±2.84	96.93±1.51	98.65±0.43	98.67±0.46
	Brain	90.12±4.77	98.78±0.91	99.34±0.41	99.28±0.43
	Prostate	91.56±2.65	94.96±2.13	97.70±1.21	97.66±1.20
2%/2 mm	H&N	81.77±4.41	91.52±2.94	94.68±2.10	94.72±2.20
	Brain	77.06±6.90	93.31±2.93	94.51±2.98	94.23±3.20
	Prostate	77.64±4.15	84.67±4.15	88.19±3.68	87.74±3.92
1%/1 mm	H&N	54.85±5.88	66.21±6.62	70.13±6.46	70.59±6.80
	Brain	45.67±7.61	62.65±8.39	64.61±9.10	64.31±9.92
	Prostate	43.23±4.95	49.32±6.20	51.18±7.14	50.43±7.55

H&N=head and neck.

### C. Percentage change in gamma passing rate

**Figure 3:**
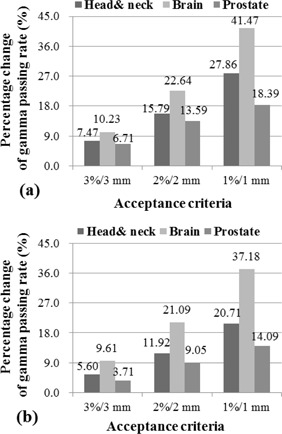
Percentage change in the gamma passing rate (%GP) compared to a low‐dose threshold of 0% for low‐dose thresholds of (a) 10% and (b) 5% in the local gamma analysis. The change in the %GP between the low‐dose thresholds of 10% and 0% is 22.64% and 10.23% for brain cancer for the 2%/2 mm and 3%/3 mm criteria, respectively (a).

The percentage change in the %GP between the low‐dose thresholds (i.e., 0%–10% and 0%–5%) was analyzed for both the global and local gamma methods, and Fig. 3 shows the results for the local normalization. In the local gamma method, the percentage change in the %GP between the low‐dose thresholds of 10% and 0% was observed to be 22.64% and 10.23% for brain cancer with the 2%/2 mm and 3%/3 mm criteria, respectively (Fig. 3(a)). The change in the %GP in the low‐dose threshold range of 0%–5% was smaller than the change in the %GP between 10% and 0% (Fig. 3(b)). All %GPs with a low‐dose threshold values above 0% were significantly different from the low‐dose threshold of 0% at the significance level of 0.0083, except for the prostate cases with a low‐dose threshold of 5% with both the 3%/3 mm and 1%/1 mm criteria. In the global gamma method, the percentage change in the %GP was lower but indicated the same tendency as in the local gamma method. However, the %GPs for the low‐dose threshold of 5%, 10%, and 15% were not significantly different than the low‐dose threshold of 0%.

### D. Gamma analysis without region of interest restriction

In the global gamma analysis, the %GP for a low‐dose threshold of 0% without the ROI constraint demonstrated the same tendency as the global gamma result, which was a decreasing trend. It showed a higher %GP than that of a low‐dose threshold of 0% with the ROI as a field. The local gamma result also indicated the same increasing tendency as the local gamma result, except for the brain cancer cases. In the local gamma analysis, the %GP was lower than that with a low‐dose threshold of 0% with the ROI as a field.

## IV. DISCUSSION

The present study was designed to determine the effect of the low‐dose threshold in gamma analysis quantitatively and to call attention to the importance of the selection of the low‐dose threshold level.

For the global gamma analysis, the %GP decreased as the low‐dose threshold increased. Our findings could be caused by the normalization method used in the gamma calculation procedure. Because the consistent maximal value was relatively higher than the dose difference in the low‐dose region, the low‐dose points usually pass the gamma analysis. The paper by Nelms et al.^(26)^ also mentioned that the global normalization of the dose difference hides errors in the lower dose regions and leads to insensitivity in the gamma analysis, especially for the 3%/3 mm acceptance criterion. Despite these facts, all cases of gamma analysis have a %GP above 95%, for both the 3%/3 mm and 2%/2 mm criteria, regardless of the applied low‐dose threshold. Thus, we concluded that applying the low‐dose threshold in global normalization does not have a critical impact on the judgment of patient‐specific QA results.

For the local gamma analysis, the %GP increased as the low‐dose threshold increased, which is the opposite result of the global gamma method. Table 4 indicates that all gamma analyses with low‐dose thresholds of 5%, 10%, and 15% for the 3%/3 mm criterion exhibited a %GP above 95% except for the prostate case with a low‐dose threshold of 5%. However, the gamma analyses with a low‐dose threshold of 0% led to a %GP below 95%. This result indicates that the low‐dose points below the dose threshold value usually fail in the local gamma analysis compared with other points. Thus, applying a low‐dose threshold causes the average %GP to increase. Figure 3 shows the percentage change in the %GP between the low‐dose threshold of 0% and low‐dose thresholds of 10% or 5%. In the local gamma analysis, the %GP with a low‐dose threshold of 10% increased by 7.47%, 10.23%, and 6.71% compared with that of the low‐dose threshold of 0% with the 3%/3 mm acceptance criterion for the head and neck, brain, and prostate cases, respectively (Fig. 3(a)). Moreover, the change in the %GP between the low‐dose thresholds of 5% and 0% ranged from 3.71% to 9.61% with the 3%/3 mm criterion (Fig. 3(b)). According to the results of the statistical analysis, the %GPs for the low‐dose threshold of 5%, 10%, and 15% were significantly different from the %GP for a low‐dose threshold of 0% except in the prostate cancer case with a low‐dose threshold of 5% for both 3%/3 mm and 1%/1 mm acceptance criteria.

Generally, the low dose occurs at the penumbra or periphery of the target; however, if the low dose is delivered to an organ at risk (OAR), it may result in fatal consequences.^27^, ^28^, ^29^ Thus, the low dose to an OAR is assessed by dose‐volume histograms (DVHs), and treatment plans are evaluated by a DVH‐based metric to restrict dose to the OAR.^27^, ^30^ Despite this effort, a low dose could lead to mortality from heart disease and lung cancer in left‐sided breast cancer radiotherapy.^(29)^ In addition, according to Dorr and Herrmann,^31^, ^32^ the majority of second cancers are induced within the penumbra of radiotherapy. Moreover, some papers reported that second cancers occurred in the low‐dose region, especially in pediatric patients.^33^, ^34^, ^35^ Thus, the low dose should be delivered accurately and evaluated strictly. However, when clinics analyze the dose distribution using gamma analysis, they usually apply the low‐dose threshold value without quantitative data.^(15)^ Our results demonstrate that the low‐dose threshold can change patient‐specific QA results in local gamma analysis because the low‐dose points excluded by applying the low‐dose threshold could affect the average %GP and increase it rapidly. Therefore, the low‐dose threshold level should be carefully applied in local gamma analysis. The Portal Dosimetry reference guide states that setting a lower dose threshold for local dose comparison is necessary because the calculated and measured values may not agree well in the low‐dose region.^(25)^ Thus, applying various low‐dose threshold levels in local gamma analysis could be a helpful approach to assess the patient‐specific QA results accurately.

For the 1%/1 mm acceptance criterion, our study showed that the average %GP exhibited a large standard deviation compared to those of the other criteria even though the average %GP and the change in the %GP between the low‐dose thresholds with 3%/3 mm, 2%/2 mm, and 1%/1 mm acceptance criteria showed a similar trend. According to several other studies, gamma analysis to detect 1% of DD and 1 mm of DTA is still controversial for the following reasons: (a) dosimetric errors and statistical fluctuations are dominant for the 1%/1 mm criterion, and (b) the acceptable level of %GP for the 1%/1 mm criterion should differ from those values for the 3%/3 mm and 2%/2 mm criteria.^36^, ^37^, ^38^ Thus, a careful approach to gamma analysis with the 1%/1 mm acceptance criterion should be taken.

To collect the results of gamma analysis, TG‐119 instructed institutions to apply a low‐dose threshold of 10% or to set the ROI as a field. Therefore, we implemented gamma analysis with the ROI set as a field to investigate the effect of the low‐dose threshold. However, we also tried to confirm whether the ROI setting affects the result, specifically the trend of the %GP. Thus, we performed additional gamma analysis without the ROI constraint. As a result, the %GP in all cases with both global and local normalization was not influenced by setting the ROI as a field. However, the local normalization of brain cancer cases was affected. The brain cancer cases showed a higher %GP than that with the field setting with a low‐dose threshold of 0%, and it was the opposite tendency from the local gamma results. This finding may be due to the fact that the brain cancer cases were planned to have a smaller field size with a narrow jaw setting, which led to an average of about 11 cm, while both the head and neck and prostate cancer cases had an average jaw setting of about 19 cm. Thus, the brain cancer cases had a larger background region that was not exposed to radiation directly and consequentially showed a higher %GP than that of the cases with a field setting and a low‐dose threshold of 0%.

## V. CONCLUSIONS

We investigated gamma analysis using various low‐dose thresholds for VMAT patient QA. For global gamma analysis, the %GP decreased with the low‐dose threshold, and the acceptance criteria of 3%/3 mm and 2%/2 mm exhibited a %GP above 95% regardless of the applied low‐dose thresholds. However, for local gamma analysis, the points excluded by the low‐dose threshold of 10% could increase the average %GP by as much as 7.47%, 10.23%, and 6.71% compared to that with a low‐dose threshold of 0% in the case of the head and neck, brain, and prostate with the 3%/3 mm criterion, respectively. Our findings suggest that the low‐dose threshold level for local gamma analysis should be carefully selected because the patient‐specific QA result of the VMAT plan can vary depending on the applied low‐dose threshold level.

## ACKNOWLEDGMENTS

This work was supported by the Radiation Technology R&D program (No. 2013M2A2A7043498) and the Mid‐career Researcher Program (2014R1A2A1A10050270) through the National Research Foundation of Korea funded by the Ministry of Science, ICT&Future Planning.

## Supporting information

Supplementary Material
