# Supplementary figures and images for: Gamma analysis dependence on specified low‐dose thresholds for VMAT QA
Source: J Appl Clin Med Phys. 2015 Nov 8;16(6):263–72. doi: 10.1120/jacmp.v16i6.5696 (PMC5691030; doi:10.1120/jacmp.v16i6.5696)

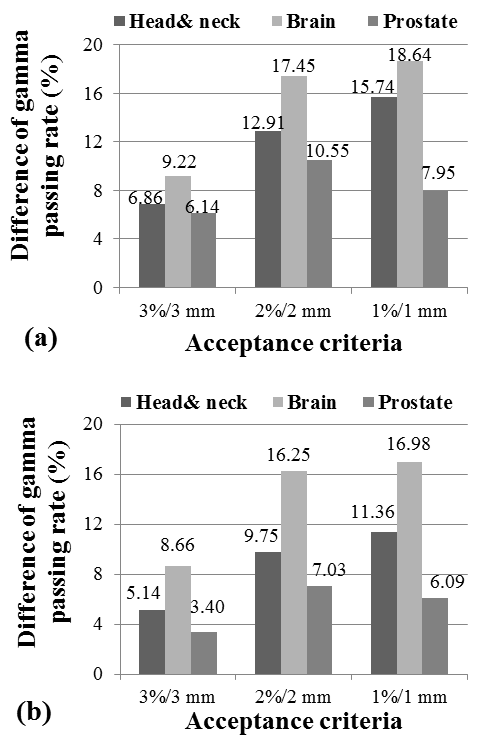

Supplement: Supplementary file 1 — Supplementary Material [file ACM2-16-263-s001.png]
